# Prevalence and Determinants of Sensitisation to Neomycin in North‐Eastern Italy, 1997–2021

**DOI:** 10.1111/cod.14730

**Published:** 2025-01-08

**Authors:** Luca Cegolon, Francesca Larese Filon, the North‐East Research Group on Contact Dermatitis

**Affiliations:** ^1^ Department of Medical, Surgical & Health Sciences University of Trieste Trieste Italy; ^2^ Public Health Department University Health Agency Giuliano‐Isontina (ASUGI) Trieste Italy; ^3^ Occupational Medicine Unit University Health Agency Giuliano‐Isontina (ASUGI) Trieste Italy; ^4^ Anna Belloni Fortina University of Padua Italy; ^5^ Erika Giulioni Azienda Sanitaria Friuli Occidentale Pordenone Italy

**Keywords:** allergic contact dermatitis, epidemiology, hapten, neomycin, patch test, prevalence, sensitisation

## Abstract

**Background:**

Allergic contact dermatitis (ACD) induced by topical application of neomycin is frequently reported.

**Objectives:**

This multi‐center study investigated prevalence and determinants of neomycin sensitisations in 30 629 outpatients from North‐eastern Italy during 1997–2021.

**Patients and Methods:**

European baseline and extended Triveneto series were applied on the upper back of patients patch testing for suspected ACD and removed after 48 h.

**Results:**

Prevalence of neomycin sensitisation was 2.29% (=701/30 629), significantly decreasing over time, especially after 2003. Neomycin sensitisation increased with age, especially in female patients older than 60 with leg dermatitis. The majority of patients sensitised to neomycin (74.5%) tested positive also against other haptens, particularly ingredients included in creams and emollients, as lanolin or benzocaine or preservatives as thimerosal or parabens.

**Conclusions:**

The decreasing prevalence of neomycin sensitisation over time likely reflected reduced accessibility and circulation of neomycin in Italy, due to containment of prescriptions and over‐the‐counter accessibility. Older individuals are typically more likely to be treated by topical medications and antibiotics as neomycin for various conditions, including stasis dermatitis. Since ACD caused by topical medications is relatively easy to miss, comprehensive drug history and patch testing are essential for any patient with suspected sensitisation caused by neomycin.

## Graphical abstract

A decreasing prevalence of neomycin sensitisation was observed over time, likely reflecting containment of prescriptions and over‐the‐counter accessibility of latter antibiotic. Since allergic contact dermatitis caused by topical medications is easy to miss, comprehensive drug history and patch testing are essential against suspected sensitisation caused by neomycin.

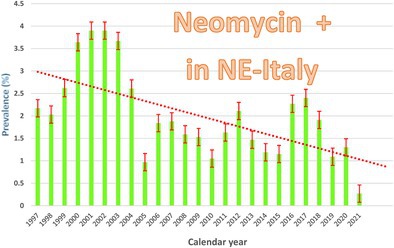

## 1 Background

Neomycin is a stable water‐soluble antibiotic of the aminoglycoside family, blocking bacterial protein synthesis by irreversibly binding with 30S ribosomal nuclear subunit [1]. Neomycin is effective against a number of anaerobic, Gram‐positive and Gram‐negative bacteria [1, 2, 3].

Neomycin indications include topical application to treat skin, ear or eye bacterial infections, solutions for urinary tract disinfections, peritoneal irrigation, treatment or prevention of infections in dentistry, veterinary, fish farming or animal feed [1, 4]. Oral administration of neomycin is also recommended to suppress intestinal bacteria before major surgery and prevent intraoperative gastrointestinal infections [1, 5].

As with other antibiotics, the main issues with neomycin intake is intolerance (nausea, vomiting, *Clostridium difficile* ‐associated colitis) or development of bacterial resistance after prolonged use [1, 2].

Allergic contact dermatitis (ACD) induced by topical medications is frequently reported in clinical practice, often misdiagnosed with infections due to inadequate clinical skill and/or poor medical history taking by health care professionals [6]. Typical presentation of ACD may be development of increased erythema, oedema, vesiculation and crusting at a surgical site 1–3 day after topical application of antibiotics as neomycin ointment [6].

Neomycin sensitisation was first reported in 1952, progressively increasing its importance as sensitiser until it was elected contact allergen of the year in 2010 [2, 7]. Although being considered a moderate sensitiser among topical medications, [8], neomycin is ranked among the top five allergens in the North American Contact Dermatitis Study Group (NACDG) [8]. Traces of neomycin can be also found in several vaccine formulations [1, 5], although the respective concentrations (25 μg) are unlikely sufficient to induce sensitisation, given higher doses (100–1000 μg) are normally needed [9, 10].

Despite limited skin and gastrointestinal absorption, systemic ACD may also rarely occur following systemic re‐exposure to neomycin in previously sensitised patients [5].

Since geographic and temporal variability of neomycin sensitisation rates is reported, mainly reflecting availability over‐the‐counter and circulation of this antibiotic [7, 10], the present study aimed to contribute and investigate prevalence and determinants of neomycin sensitisations in North‐eastern Italy during 1997–2021 (25 years).

## 2 Methods

This multicentric cross‐sectional study aimed to investigate the prevalence of CD induced by neomycin in a cohort of 30 629 patients patch tested during 1997–2021 (25 years) in various outpatients from North‐Eastern Italy.

This study was approved by the local ethical committee of Friuli Venezia Giulia (CEUR, protocol 092/2018), and written informed consent was obtained from all participating patients.

### 2.1 Study Population

The ‘Triveneto patch database’ includes information on patch tests consecutive performed between 1997 and 2021 in various outpatient services of North‐Eastern Italy. Whilst the Triveneto series varied over the years, the 22 haptens displayed in Table S1 were consistently tested during the entire study period.

### 2.2 Patients' Evaluation

The clinical pattern of patients was assessed using the MOAHLFA Index (considering sex of patient, occupational dermatitis, atopic dermatitis, hand involvement, leg involvement, face involvement and age > 40 years) [11]. Occupational CD was assessed by a dermatologist or an occupational medicine consultant. Atopic eczema was defined according to clinical history, asking patients whether they suffered from atopic eczema/eczema in flexures currently or during childhood. Prevalence of sensitisation to neomycin was monitored over time in order to identify potential trends.

All patients were patch tested with Finn Chambers (Epitest, Tuusula, Finland) on Scanpor tape (Norgesplaster, Vennesla, Norway) and haptens produced by Chemotechnique Diagnostics (Vellinge, Sweden) and by FIRMA (Florence, Italy). European baseline series and the extended Triveneto series were used to patch test patients with suspected CD. All patches were applied on the upper part of patient back and removed after 48 h. The area was examined upon removal of the patch (D2) and after 72/96 h (D3/D4), according to guidelines of the International Contact Dermatitis Research Group [12]:

- Reactions degree +, ++ and +++ were considered positive.
- Uncertain responses (?+) were considered negative.

### 2.3 Statistical Analysis

Continuous variables were presented as mean and standard deviation. Medians were contrasted by Wilcoxon test, whereas the Chi‐squared test was employed to compare categorical variables.

Using administrative clerks as reference, univariable and multivariable logistic regression analysis was employed to investigate the risk of sensitisation to neomycin controlling for a number of explanatory variables, reporting odds ratio unadjusted (OR) and adjusted (aOR) with 95% confidence interval (95% CI).

Concurrent sensitisation was investigated using multivariable logistic regression adjusting for age and sex.

The statistical analysis of the data was performed with STATA version 14.0 (Stata, College Station, TX, USA).

## 3 Results

Thirty thousand six hundred twenty‐nine patients with suspected ACD were patch tested against neomycin and a total of 701 positive reactions against the latter hapten were detected.

**FIGURE 1:**
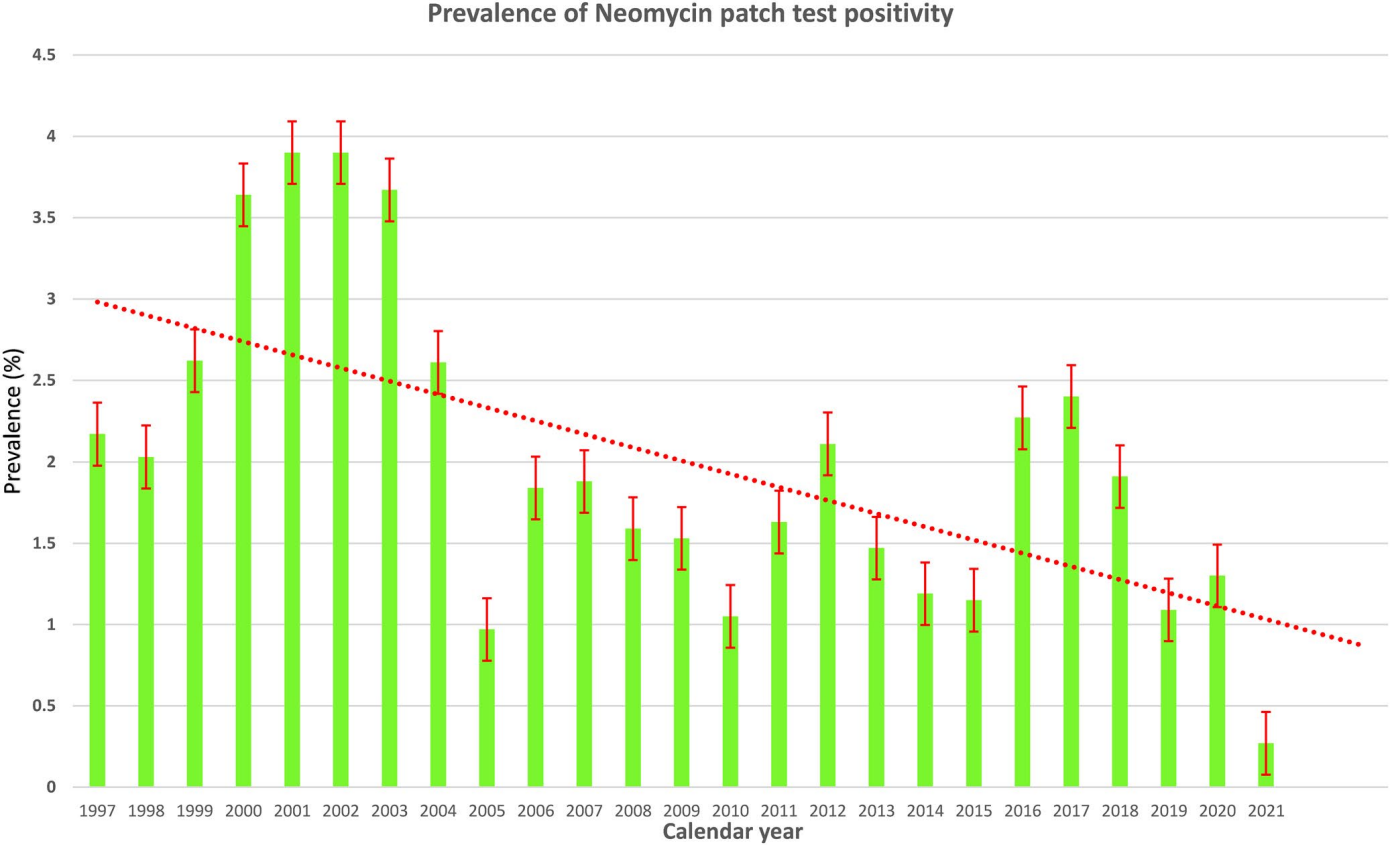
Prevalence of patch test positivity to neomycin over time (1997–2021), with trend‐line. Standard error bars are red marke.

Table S2 and Figure 1 display the temporal trend of neomycin positive patch tests in the study population. As can be seen, neomycin sensitisation increased sharply during years 2001–2005, significantly decreasing thereafter. Twenty‐five‐point‐five % of patients mono‐sensitised at patch test, 27.2% reacted against one additional hapten and 21.9% against two.

As can be appreciated from Figure  and Table 1, prevalence of neomycin sensitisation was lower in the Provinces of Trento‐Bolzano/Rovigo (0.99%) or Pordenone (1.35%).

**TABLE 1:** Distribution of the study population by percentage of neomycin sensitisation and univariable as well as multivariable logistic regression analysis for the risk sensitisation to neomycin.

Terms			Total patients Patch tested *N* (col %)	Neomycin + *N* (row %)	*p*	OR (95% CI)	aOR (95% CI) (26 049 obs.)
Total patients examined for CD			**30 629 (100)**	**701 (2.29)**			
Centre	Padua		9562 (31.22)	267 (2.79)	< 0.001	Reference	Reference
	Pordenone		7471 (24.39)	101 (1.35)		0.48 (0.38; 0.60)	0.55 (0.43; 0.71)
	Trieste		9963 (32.53)	297 (2.98)		1.07 (0.90; 1.27)	1.21 (0.99; 1.48)
	Trento/Bolzano/Rovigo		3363 (11.86)	36 (0.99)		0.35 (0.25; 0.49)	0.30 (0.20; 0.45)
Sex	Females		20 694 (67.56)	510 (2.46)	0.003	Reference	Reference
	Males		9935 (32.44)	191 (1.92)		0.78 (0.66; 0.92)	0.79 (0.64; 0.96)
Age (years) (M: 3)	M ± SD		43.79 ± 17.24	50.2 ± 17.7			
	Median		42 (30; 57)	50 (36; 65)	< 0.001		
	< 41		14 522 (47.42)	239 (1.65)	< 0.001	Reference	Reference
	40+		16 104 (52.58)	462 (2.87)		1.77 (1.51; 2.07)	1.55 (1.27; 1.91)
Atopic dermatiis (M: 3143)	No		24 718 (89.93)	578 (2.34)	0.197	Reference	
	Yes		2768 (10.07)	54 (1.95)		0.83 (0.63; 1.10)	
Occupational dermatitis (M: 31)	No		28 078 (91.76)	657 (2.34)	0.042	Reference	
	Yes		2520 (8.24)	43 (1.71)		0.72 (0.53; 0.99)	
Body area affected by CD	Hand (M: 4432)	No	16 683 (63.68)	423 (2.54)	< 0.001	Reference	
		Yes	9514 (36.32)	165 (1.73)		0.68 (0.57; 0.81)	
	Leg (M: 4430)	No	24 080 (91.91)	514 (2.13)	< 0.001	Reference	Reference
		Yes	2119 (8.09)	74 (3.49)		1.66 (1.29; 2.13)	1.60 (1.24; 2.06)
	Face (M: 4430)	No	21 084 (80.48)	461 (2.19)	0.199	Reference	Reference
		Yes	5115 (19.52)	127 (2.48)		1.13 (0.93; 1.39)	1.33 (1.08; 1.64)
Calendar year (1996–2022)	Linear term (1997–2021)				< 0.001	0.96 (0.95; 0.97)	0.94 (0.93; 0.96)
	1997–1998		3808 (12.43)	79 (2.07)	< 0.001	Referenc*e*	
	1999–2004		11 499 (37.54)	391 (3.40)		1.66 (1.30; 2.12)	
	2005–2012		8468 (27.65)	131 (1.55)		0.74 (0.56; 0.98)	
	2013–2021		6854 (22.38)	100 (1.46)		0.70 (0.52; 0.94)	

*Note*: Number (*N*), row percentage (%); chi square *p* value; Odds ratio unadjusted (OR) and adjusted (aOR) with 95% confidence interval (95% CI). Multivariable model adjusted also for occupation (Table S3). Light green = OR < 1 and > 80; green = protective effect OR < 0.80; light yellow = OR < 2; yellow = OR 2–3; dark yellow = OR 3–4; orange = > 4.

Abbreviations: CD, contact dermatitis; M, missing values; Obs., complete case (analysis) observations.

Table 1 displays the distribution of study population by prevalence of neomycin sensitisation and explanatory factor, reporting also the results of univariable as well as multivariable logistic regression analysis. As can be noted, 701 (2.29%) patients tested positive for neomycin out of 30 629 study subjects examined and patch tested for suspected ACD.

The mean age of patients testing positive for neomycin was 50.2 ± 17.7 years and 72.7% (=510/701) were females (Table 1).

Atopic dermatitis was identified in 2768 (10.1%) patients, with 1.95% (=54/2768) of atopic patients testing positive against neomycin. Prevalence of occupational CD was 8.2%, with 1.7% (=43/2520) patients with occupational CD patch testing positive against neomycin (Table 1).

The body areas most frequently affected by CD in those patients undertaking patch test for neomycin were hands (36.3%), followed by face (19.5%) and legs (8.1%), with a 1.7% testing positive at patch test, 3.5% and 2.5%, respectively (Table 1).

At multiple logistic regression the risk of sensitisation to neomycin was significantly lower in Trento–Bolzano–Rovigo (aOR = 0.30; 95% CI: 0.20; 0.45) or Pordenone (aOR = 0.55; 95% CI: 0.43; 0.71) and among males (aOR = 0.79; 95% CI: 0.64; 0.96).

Moreover, prevalence of neomycin sensitisation was significantly higher in patients aged 40+ years (aOR = 1.55; 95% CI: 1.27; 1.91), those with face CD (aOR = 1.60; 95% CI: 1.24; 2.06) or leg (aOR = 1.33; 95% CI: 1.08; 1.64) CD.

Compared to administrative clerks, sensitisation was significantly higher in retirees (aOR = 1.40; 95% CI: 1.06; 1.83) (Table S3).

A significantly decreasing trend over time of neomycin sensitisation was observed during 1997–2021 (aOR = 0.94; 95% CI: 0.93; 0.96) (Table 2).

Table 2 shows a further multiple logistic regression model fitted on the entire cohort and by sex of patients, adjusting for center, age, sex, leg CD, calendar year and occupation, but including an interaction term between age and leg CD. As can be seen, prevalence of neomycin sensitisation significantly decreased over time (aOR = 0.94; 95% CI: 0.93; 0.96) and in males (aOR = 0.76; 95% CI: 0.63; 0.93), yet it increased in patients older than 38 years, especially in those aged 61+ years. However, the age trend was remarkably stronger for patients with leg CD, especially 61+ years of age (aOR = 3.75; 95% CI: 2.32; 6.05). Sub‐group analysis by sex of patients confirmed the above findings, with stronger and more clear effect of age in females 61+ years than males, without (aOR = 2.66; 95% CI; 1.72; 4.12) or even more with (aOR = 4.16; 95% CI 2.33; 7.40) leg CD. The higher risk of neomycin sensitisation in retirees lost statistical significance with this analysis (data not shown).

**TABLE 2:** Distribution of study population by percentage of neomycin sensitisation and univariable as well as multivariable logistic regression analysis for the risk sensitisation to neomycin.

Terms				All patients		Males		Females	
				Neomycin + *N* (row %)	aOR (95% CI) (26 049 obs.)	Neomycin + *N* (row %)	aOR (95% CI) (8318 obs.)	Neomycin + *N* (row %)	aOR (95% CI) (17 604 obs.)
Sex		Females		501 (2.46)	Reference				
		Males		191 (1.92)	0.76 (0.63; 0.93)				
Leg CD (Interaction)	No	Age (years)	< 28	69 (1.35)	Reference	17 (1.07)	Reference	52 (1.47)	Reference
			27–37	82 (1.63)	1.27 (0.90; 1.77)	19 (1.16)	1.17 (0.59; 2.32)	63 (1.85)	1.31 (0.89; 1.93)
			38–48	102 (2.08)	1.73 (1.25; 2.40)	19 (1.29)	1.38 (0.69; 2.77)	83 (2.42)	1.86 (1.28; 2.70)
			49–60	111 (2.45)	1.98 (1.42; 2.77)	35 (2.43)	2.43 (1.29; 4.58)	76 (2.45)	1.83 (1.24; 2.71)
			61+	150 (3.36)	2.54 (1.75; 3.70)	46 (3.14)	2.43 (1.17; 5.06)	184 (3.46)	2.66 (1.72; 4.12)
	Yes	Age (years)	< 28	7 (2.30)	1.66 (0.75; 3.65)	1 (0.85)	0.85 (0.11; 6.54)	6 (3.19)	1.98 (0.83; 4.71)
			27–37	7 (2.31)	1.86 (0.84; 4.13)	2 (1.85)	1.75 (0.39: 7.89)	5 (2.56)	1.89 (0.74; 4.86)
			38–48	14 (3.58)	3.19 (1.76; 5.81)	5 (3.33)	3.80 (1.34; 10.75)	9 (3.73)	2.95 (1.41; 6.19)
			49–60	10 (2.56)	2.21 (1.11; 4.40)	6 (3.17)	3.13 (1.17; 8.38)	4 (1.99)	1.55 (0.54; 4.40)
			61+	36 (4.93)	3.75 (2.32; 6.05)	13 (4.01)	3.12 (1.31; 7.45)	23 (5.67)	4.16 (2.33; 7.40)
Year 1997–2021					0.94 (0.93; 0.96)		0.95 (0.92; 0.97)		0.92 (0.93; 0.96)

*Note*: Number (*N*), row percentage (%) and chi square *p* value. Odds ratio unadjusted (OR) and adjusted (aOR) with 95% confidence interval (95% CI). All 3 regression models were adjusted for research center, sex, age, leg CD; occupation and calendar year. Light green = OR < 1 and > 80; green = protective effect OR < 0.80; light yellow = OR < 2; yellow = OR 2–3; dark yellow = OR 3–4; orange = > 4.

Abbreviations: CD, contact dermatitis; M, missing values; Obs., complete case (analysis) observations.

Table 3 reports the number and percentage of sensitizations to neomycin sulphate 20% and ohter haptens, with logistic regression models adjusting for age and sex. Stronger associations were found for ingredients of creams and emollients, as lanolin alcohol 30% pet. (OR = 4.18; 95% CI 3.04–5.73), benzocaine (OR = 2.61; 95% IC 1.54–4.43), preservatives as thimerosal (OR = 2.05; 95% CI 1.58–2.68), parabens (OR = 2.73; 95% CI1.70–4.39), perfumes such as Fragrance Mix I (OR = 1.62; 95% CI 1.29–2.05) or Balsam of Peru (OR = 1.97; 95% CI 1.56–2.49). Co‐sensitisations were also more likely against other allergens such as metals (nickel, cobalt and chromium), dyes (p‐phenylendiamine, disperse blu 124), resins (colophonium, PTBP‐FR and epoxy resin), rubber additives and other chemicals (carba mix, IPPD and diaminodiphenylmethane).

**TABLE 3:** Concurrent sensitisation between neomycin sulphate 20% and other haptens (all in pet, except where indicated otherwise).

HAPTENS	Neomycin − *N* (%) Tot = 29 927	Neomycin + *N* (%) Tot = 701	aOR (95% CI)	*p*
Balsam of Peru (myroxylon pereirae) 25%	493 (1.65)	14 (2.0)	1.97 (1.56; 2.49)	< 0.001
Benzocaine 5%	233 (0.80)	15 (2.19)	2.61 (1.54; 4.43)	< 0.001
Carba mix	1038 (3.47)	48 (6.85)	2.13 (1.57; 2.88)	< 0.001
Colophonium 20%	500 (1.67)	24 (3.42)	2.01 (1.32; 3.05)	0.001
Cobalt chroride hexahydrate 1%	2748 (9.18)	130 (18.5)	2.38 (1.95; 2.89)	< 0.001
Diaminodiphenylmethane 0.5%	690 (2.31)	45 (6.42)	2.62 (1.92; 3.58)	< 0.001
Disperse blu 124 1%	743 (2.48)	37 (5.28)	2.15 (1.53; 3.02)	< 0.001
Epoxy resin bisphenol A 1%	237 (0.79)	10 (1.43)	1.95 (1.03; 3.69)	0.001
Fragrance mix I	2117 (7.07)	84 (11.98)	1.62 (1.29; 2.05)	< 0.001
IPPD 0.1%	227 (0.76)	18 (2.57)	3.28 (2.01; 5.35)	< 0.001
Lanolin alcohol 30%	455 (1.52)	46 (6.56)	4.18 (3.04; 5.73)	< 0.001
Nickel sulphate 5%	7838 (28.19)	234 (33.38)	1.56 (1.32; 1.84)	< 0.001
Paraben mix	285 (0.95)	19 (2.71)	2.73 (1.70; 4.39)	< 0.001
p‐Phenylendiamine 0.5%	1056 (3.53)	40 (5.71)	1.57 (1.13; 2.18)	0.007
PTBP‐FR 1%	325 (1.09)	16 (2.28)	2.14 (1.29; 3.56)	0.003
Potassium dichromate 0.5%	1922 (6.42)	127 (18.12)	3.22 (2.64; 3.93)	< 0.001
Thimerosal 0.1% aq	1753 (5.99)	63 (9.45)	2.05 (1.58; 2.68)	< 0.001

*Note*: Multiple logistic regression models for the risk of co‐sensitisation with neomycin, adjusted for age and sex. Number (*N*), row percentage (%), adjusted odds ratio (aOR) with 95% confidence interval (95% CI). Only significant associations are reported.

Abbreviations: IPPD, N.isopropyl‐N‐phenyl‐4‐phenylendiamine; PTBP‐FR, para‐tertiary‐butyl phenol formaldhyde resin.

## 4 Discussion

### 4.1 Prevalence of Neomycin Sensitisation

The overall prevalence of patients testing positive against neomycin (2.29%) of the present study, significantly decreasing over time, is in line with corresponding figures from Europe. Neomycin sensitisation varied in fact between 1.1% and 3.8%, averaging around 2.6% annually in Europe, according to a meta‐analysis of studies conducted in major European centres during 1996–2006 [2, 13]. Likewise, among 47 559 patients referring to the Information Network Departments of Dermatology (IVDK) from Germany, Austria and Switzerland, the prevalence of neomycin sensitisation was 2.5% during years 1998–2003 [14] and 2.9% according to another multi‐center study on 9672 patients from 9 European countries patch tested during 2002–2003 [15]. More recent data from the European Surveillance System for Contact Dermatitis (ESSCA) group reported a prevalence of neomycin sensitisation of 1.32% in 2009–2012 [16], 1.23% in 2015–2018 [17] and 0.83% in 2019–2020 [12]. A cross‐section study on 17 849 patients consecutively patch‐tested at Gentofte Hospital (Denmark) during 2000–2023, prevalence of neomycin sensitisation was 1.4% [18].

Differences by centres in the present study could be attributable to higher use of topical antibiotics against skin infections in larger cities as Padua and Trieste, compared to more rural areas in Pordenone or Trento/Bolzano/Rovigo.

As mentioned above, prevalence of neomycin sensitisation was higher in the past in Europe. For instance, in the early 70ies the proportion of patch test positive reactions was 6.9% among 1312 patients from Western Scotland and 5.1% in 450 patients from S. John's Hospital in London [19], increasing from 5% in 1990 to 10.2% in 1992 in Croatia [20] and being 18.4% among 562 children patch tested in Portugal in 1991 [21]. Prevalence of positive patch tests significantly decreased from 8.2% in 1995–1996 to 5.4% in 2000–2002 in a multi‐centre study covering 7 dermatological clinics affiliated to the Finnish Contact Dermatitis Group [22] and was 3.9% in a UK study comparing frequency of ACD against topical antibiotics in 1119 patients patch tested at S. John's Institute of Dermatology in London in 1998 [8].

The diminishing sensitisation trend over time observed in Europe may be largely attributable to contained accessibility and circulation of neomycin medications [7], since over‐the‐counter antibiotics are reportedly more sensitising than prescribed ones [23].

Despite being higher in North America (7%–13%) during the past two decades [24, 25, 26, 27, 28, 29], prevalence of neomycin sensitisation rate decreased from 7.37% during 2001–2008 to 1.73% in 2009–2013 in Western Canada and was 3.2% among 821 patients patch tested at St Paul's Hospital in Vancouver between November 2016 and June 2019 [6]. By contrast, during 2000–2010, neomycin was the second most common topical medication inducing ACD after bacitracin at Ottawa patch test clinic [30]. Again, declining sensitisation over time, aligning Canada with Europe, likely reflects implementation of policies to contain accessibility over‐the‐counter of neomycin‐containing medications [7], now available only by prescription in Canada [6].

By contrast, in the USA, the average neomycin sensitisation rate was 11.4% during 1996–2006 [10], considerably higher than in the 70ies, when the proportion of patients testing positive was reportedly 6.4% in 1974–75 and 5.6% in 1975–1976 [29]. Sensitisation to neomycin was 10% during 2005–2006 according to a NACDG study, ranking this antibiotic 5th among 15 most common allergens [26]. More recently, the rate of neomycin patch test reactions increased even more (11.7%) in another NACDG study on 28 640 patients patch tested during 2005–2016, with neomycin being the third most prevalent hapten in the USA, after Nickel (16.0%) and methylisothiazolinone (13.4%) [31]. Finally, in a study on 2373 patients patch tested at Massachusetts General Hospital during 2007–2016, neomycin sulphate (9.4%) was the fourth most prevalent hapten after nickel sulphate (19.8%), fragrance mix (14.6%) and Balsam of Peru (13.5%). Previous prevalence estimates of neomycin sensitisation rates in the same institution were 4.8% during 1990–2006 and 4.6% during 1998–2006 [32].

### 4.2 Risk Factors for Neomycin Sensitisation

Males were less likely to test positive against neomycin in the present study and prevalence of sensitisation was remarkably higher in females older than 60, especially among those affected by leg CD. Likewise, in Western Canada during 2001–2013 females were more likely to patch test positive (RR = 2.75; 95% CI: 1.86; 4.06) [7]. Females may be more prone to use topical treatments. In the present study, prevalence of neomycin sensitisation increased with age, regardless of presence of leg CD and the effect of age prevailed over retirement occupation at multiple regression analysis. Likewise, patients older than 40 years were consistently at higher risk (OR = 1.30; 95% CI =1.22; 1.38) of sensitisation against neomycin in the above NACDG study on 28 640 patients patch tested during 2005–2016 in the USA [31]. Leg and face were body districts more likely to be associated with neomycin sensitisation in the present study. It can be reasonably argued that older individuals may be more likely to be treated by topical antibiotics as neomycin for various conditions, including stasis dermatitis [8].

We did not find any association between occupation and neomycin sensitisation, endorsing a prevalent extra‐occupational exposure to this antibiotic. Although health care workers, pharmacists, dentists and veterinarians may be exposed to neomycin when handling topical antibiotics, mandatory use of gloves prevents direct skin contact with creams and ointments containing haptens [2].

### 4.3 Co‐Sensitisations and Clinical Recommendations

ACD is a type IV delayed hypersensitivity reaction presenting with an itching eczematous rash developing after direct skin contact with a hapten. A previous sensitisation event is necessary, at least 2 weeks before [6]. A single topical application may be sufficient to induce sensitisation, an event easily neglected at medical history taking [6].

Patch testing to a topical antibiotic and its components contribute to identify specific allergens, although the sensitisation may not be against the commercial formulation but to any individual ingredient [6]. Topical medications (ointments, eye drops, ear drops, powers, etc.) may share components potentially causing side effects, including ACD, irritative CD or urticaria [7]. Co‐sensitisation, a frequent phenomenon with topical antibiotics [30], is defined as ACD to different components included in the same source, whereas cross‐sensitisation is ACD against 2+ different haptens sharing a similar chemical structure but coming from different sources [6].

In the present study the majority of patients sensitised to neomycin (74.5%) tested positive also against other haptens, particularly ingredients of creams and emollients as lanolin or benzocaine or preservatives as thimerosal or parabens. It is therefore hard to disentangle the role of neomycin in a context of polysensitisation to various haptens that can be potentially found in skin products and emollients.

In the study on 821 patients patch tested at St Paul's Hospital in Vancouver, the three active principles with highest prevalence of co‐sensitisation were neomycin (22.9%), bacitracin (20.0%) and polymyxin B (18.6%) [6]. Other studies also reported high levels of antibiotic co‐sensitisation [30]. Cross‐sensitisation was described in a patient with known ACD to neomycin developing erythroderma following gentamicin iv [6, 33]. Deoxystreptamine class of aminoglycoside antibiotics reportedly exhibit a 50% prevalence of cross‐reactivity [34]. The cross‐reactivity among neomycin and other aminoglycosides is well known [1] and involve amikacin, framycetin, gentamycin, paromycin, sisomicin and tobramycin used in clinical dermatology [18].

Careful drug history taking, often overlooked, is therefore necessary not only under suspicion of dermatitis caused by topical medications [7] but also in view of possible co‐sensitisations and/or cross‐reactions [6].

Moreover, in order to contain sensitisation against neomycin, alternative medications as petrolatum or other emollients—not enhancing the risk of sensitisation—are recommended for post‐operative wound care [35, 36, 37, 38, 39].

#### 4.3.1 Strength and Weaknesses

The present is the largest study investigating neomycin sensitisation in Italy over a long time period (25 years), employing a multi‐centre data collection, assessing also the impact of occupation and yielding adjusted prevalence estimates over time. However, study limitations include the cross‐sectional design, the variability of testing prevalence by research centre and calendar year and the lack of definition of clinical relevance for neomycin sensitisation. However, avoidance of assessment of clinical relevance, as done in other epidemiological studies [40, 41], reduced the impact potential interpretation bias.

Moreover, the reading was done for the majority of cases at 96 h since the start of the database. The reading at 72 h was performed in Trieste in about a fourth of patients due to organisational and technical constraints. However, patients were still recommended to come back to be seen in ambulatory in the event of late reactions onset.

## 5 Conclusions

Overall prevalence of neomycin sensitisation was 2.29% in the present study.

The decreasing sensitisation rates over time likely reflected reduced circulation of neomycin in Italy as in the rest of Europe, due to containment of prescriptions and over‐the‐counter accessibility.

Sensitisation to neomycin increased with age, especially in female patients with leg CD. Older individuals are more likely to be treated by topical medications and antibiotics as neomycin for various conditions, including stasis dermatitis. Antibiotics as neomycin are the most frequent cause of ACD induced by topical medications, even in the form of co‐sensitisation. Since it is relatively easy to miss, ACD should be consistently considered for differential diagnosis in case of any eczematous rash. Comprehensive drug history and patch testing are recommended for any patient with suspected ACD against topical antibiotics.

## Author Contributions

**Luca Cegolon:** formal analysis, writing – original draft, investigation, methodology. **Francesca Larese Filon:** writing – review and editing, supervision, methodology, conceptualization.

## Consent

This study was approved by the local ethical committee of Friuli Venezia Giulia (CUER, protocol 092/2018) and written informed consent was obtained from all participating patients.

## Conflicts of Interest

The authors declare no conflicts of interest.

## Supporting information


Data S1.

## Data Availability

The data generated and analysed during the current study are not publicly available since they were purposively collected by the authors for the present study, but they are available from the corresponding author upon reasonable request.
