# Supplementary material for: Prevalence and Determinants of Sensitisation to Neomycin in North‐Eastern Italy, 1997–2021
Source: Contact Dermatitis. 2025 Jan 8;92(6):460–8. doi: 10.1111/cod.14730 (PMC12055313; doi:10.1111/cod.14730)
Supplement: Supplementary file 1 — Data S1. [file COD-92-460-s001.docx]

**Supplementary Table 1.** Triveneto patch test series (22 haptens) tested in the overall study period (all in pet when not otherwise specified)

| **1** | 4-ter-Butylphenol- formaldehyde resin 1% |
| --- | --- |
| **2** | Carba mix 3% |
| **3** | Cobalt chloride hexahydrate 1% |
| **4** | Colophonium 20% |
| **5** | Disperse blu 35 1% |
| **6** | Disperse yellow 3 1% |
| **7** | Epoxy resin 1% |
| **8** | Formaldehyde 1% aq |
| **9** | Fragrance mix-I 8% |
| **10** | Methyl-chloro-isothiazolinon/methyl-isothiazolinon (Kathon) 0.02 aq. |
| **11** | Lanolin alcohol 30%, |
| **12** | Mercaptobenzothiazole 2% |
| **13** | Mercaptobenzothiazole mix |
| **14** | Neomycin sulfate 20% |
| **15** | Nickel sulphate 5% |
| **16** | N-Isopropy-N-phenyl-4-phenylendiamine 0.1% |
| **17** | Parabens mix |
| **18** | Peru balsam 25% |
| **19** | Potassium bichromate 0.5% |
| **20** | p-Phenylenediamine 1% |
| **21** | Quaternium-15 1% |
| **22** | Thiuram mix 1% |

| **Supplementary Table 2.** Frequency distribution of patients patch tested for contact dermatitis and rates of positivity against Neomycin by calendar year (1997-2021) and research centre. Number (N) and row percentage (%) | | | | | | | | | | |
| --- | --- | --- | --- | --- | --- | --- | --- | --- | --- | --- |
| **CALENDAR**  **YEAR** | **ENTIRE**  **COHORT** | | **RESEARCH CENTRE** | | | | | | | |
|  |  |  | **Padua** | | **Pordenone** | | **Trieste** | | **Trento/Bolzano/Rovigo** | |
|  | **N. tests** | **Neomycin +** | **N. tests** | **Neomycin +** | **N. tests** | **Neomycin +** | **N. tests** | **Neomycin +** | **N. tests** | **Neomycin +** |
| **1997** | 1,242 | 27 (2.17) | 395 | 17 (4.30) | 445 | 4 (0.90) | 398 | 6 (1.51) | 4 | 0 |
| **1998** | 2,566 | 52 (2.03) | 1,083 | 32 (2.95) | 325 | 0 | 418 | 13 (3.11) | 740 | 7 (0.95) |
| **1999** | 2,868 | 75 (2.62) | 1,189 | 41 (3.45) | 278 | 1 (0.36) | 453 | 27 (5.96) | 948 | 6 (0.63) |
| **2000** | 2,445 | 89 (3.64) | 1,042 | 34 (3.26) | 208 | 3 (1.44) | 817 | 49 (6.00) | 378 | 3 (0.79) |
| **2001** | 2,412 | 94 (3.90) | 677 | 26 (3.84) | 226 | 4 (1.77) | 703 | 53 (7.54) | 806 | 11 (1.36) |
| **2002** | 1,460 | 57 (3.90) | 1 | 0 | 353 | 7 (1.98) | 701 | 43 (6.13) | 405 | 7 (1.73) |
| **2003** | 1,472 | 54 (3.67) | 412 | 31 (7.52) | 314 | 2 (0.64) | 485 | 21 (4.33) | 261 | 0 |
| **2004** | 842 | 22 (2.61) | 287 | 7 (2.44) | 94 | 2 (2.13) | 370 | 11 (2.97) | 91 | 2 (2.20) |
| **2005** | 1,135 | 11 (0.97) | 434 | 3 (0.69) | 383 | 4 (1.04) | 318 | 4 (1.26) | 0 | NA |
| **2006** | 1,032 | 19 (1.84) | 409 | 10 (2.44) | 336 | 2 (0.60) | 287 | 7 (2.44) | 0 | NA |
| **2007** | 1,115 | 21 (1.88) | 439 | 14 (3.19) | 368 | 5 (1.36) | 308 | 2 (0.65) | 0 | NA |
| **2008** | 1,257 | 20 (1.59) | 528 | 9 (1.70) | 355 | 5 (1.41) | 374 | 6 (1.60) | 0 | NA |
| **2009** | 1,112 | 17 (1.53) | 328 | 5 (1.52) | 373 | 7 (1.88) | 411 | 5 (1.22) | 0 | NA |
| **2010** | 1,046 | 11 (1.05) | 354 | 7 (1.98) | 366 | 2 (0.55) | 326 | 2 (0.61) | 0 | NA |
| **2011** | 1,107 | 18 (1.63) | 234 | 5 (2.14) | 377 | 7 (1.86) | 496 | 6 (1.21) | 0 | NA |
| **2012** | 664 | 14 (2.11) | 0 | 0 | 286 | 3 (1.05) | 378 | 11 (2.91) | 0 | NA |
| **2013** | 886 | 13 (1.47) | 240 | 1 (0.42) | 274 | 6 (2.19) | 372 | 6 (1.61) | 0 | NA |
| **2014** | 1,011 | 12 (1.19) | 322 | 2 (0.62) | 288 | 4 (1.39) | 401 | 6 (1.50) | 0 | NA |
| **2015** | 958 | 11 (1.15) | 246 | 3 (1.22) | 327 | 4 (1.22) | 385 | 4 (1.04) | 0 | NA |
| **2016** | 749 | 17 (2.27) | 230 | 6 (2.61) | 239 | 6 (2.51) | 280 | 5 (1.79) | 0 | NA |
| **2017** | 501 | 12 (2.40) | 102 | 1 (0.98) | 241 | 8 (3.32) | 158 | 3 (1.90) | 0 | NA |
| **2018** | 836 | 16 (1.91) | 135 | 7 (5.19) | 451 | 7 (1.55) | 250 | 2 (0.80) | 0 | NA |
| **2019** | 1,008 | 11 (1.09) | 381 | 4 (1.05) | 211 | 4 (1.90) | 416 | 3 (0.72) | 0 | NA |
| **2020** | 540 | 7 (1.30) | 94 | 2 (2.13) | 265 | 4 (1.51) | 181 | 1 (0.55) | 0 | NA |
| **2021** | 365 | 1 (0.27) | 0 | 0 | 88 | 0 | 277 | 1 (0.36) | 0 | NA |
| **Total** | **30,629** | **701 (2.29)** | **267 (2.79)** | **7,741** | **191** | **101 (1.35)** | **9,963** | **197 (2.98)** | **3,633** | **36 (0.99)** |

**Supplementary Figure 1**. Prevalence of patch tests positivity against Neomycin during 1997-2021, by research center (“*Triveneto patch database*”).

| **Supplementary Table 3.**  Distribution of the study population by percentage of neomycin sensitization and univariable as well as multivariable logistic regression analysis for the risk sensitization to neomycin. Number (N), row percentage (%); chi square p-value; Odds ratio unadjusted (OR) and adjusted (aOR) with 95% confidence interval (95%CI). CD= contact dermatitis; M=missing values. Obs.= complete case (analysis) observations. Multivariable model adjusted for the same factors as in **Table 1** (center, age, sex, leg CD, face, CD, calendar year, occupation). Orange highlights denote significantly increased risk of sensitization; green highlights denote significantly reduced risk of sensitization | | | | | | |
| --- | --- | --- | --- | --- | --- | --- |
| **Terms** | **Categories** | **Total patients with CD**  **N (col %)** | **Neomycin +**  **N (row %)** | **p-value** | **OR (95%CI)** | **aOR (95%CI)**  (26,049 obs.) |
| **Occupation** | **Clerks** | 6,692 (21.85) | 144 (2.15) | < 0.001 | *reference* | *reference* |
|  | **Health care workers** | 3,087 (10.08) | 63 (2.04) |  | 0.95 (0.70; 1.28) | 0.85 (0.61; 1.19) |
|  | **Teachers** | 364 (1.19) | 4 (1.10) |  | 0.51 (0.19; 1.37) | 0.66 (0.24; 1.82) |
|  | **Cashiers** | 26 (0.08) | 0 |  | 1 | 1 |
|  | **Sellers** | 353 (1.15) | 5 (1.42) |  | 0.65 (0.27; 1.60) | 0.83 (0.34; 2.06) |
|  | **Restaurant workers** | 1,297 (4.23) | 27 (2.08) |  | 0.97 (0.64; 1.46) | 1.12 (0.71; 1.74) |
|  | **Hair-dressers** | 388 (1.27) | 3 (0.77) |  | 0.35 (0.11; 1.12) | 0.49 (0.15; 1.56) |
|  | **Farmers** | 257 (0. 84) | 7 (2.72) |  | 1.27 (0.59; 2.75) | 1.15 (0.46; 2.85) |
|  | **Construction workers** | 1,178 (3.85) | 24 (2.04) |  | 0.95 (0.61; 1.46) | 0.82 (0.50; 1.37) |
|  | **House painter** | 26 (0.08) | 1 (3.85) |  | 1.82 (0.24; 13.52) | 2.66 (0.35; 20.12) |
|  | **Painter other** | 76 (0.25) | 2 (2.63) |  | 1.23 (0.30; 5.05) | 1.38 (0.33; 5.74) |
|  | **Construction cleaners** | 17 (0.06) | 0 |  | 1 | 1 |
|  | **Mechanics** | 1,485 (4.85) | 20 (1.35) |  | 0.62 (0.39; 0.99) | 0.59 (0.33; 1.06) |
|  | **Workers of wood industry** | 440 (1.44) | 7 (1.59) |  | 0.74 (0.34; 1.58) | 0.76 (0. 31; 1.87) |
|  | **Artisan general** | 454 (1.48) | 10 (2.20) |  | 1.02 (0.54;1.96) | 1.12 (0.56; 2.23) |
|  | **Leather artisans** | 114 (0.37) | 1 (0.88) |  | 0.40 (0.06; 2.90) | 1 |
|  | **Chemistry Industry workers** | 229 (0.75) | 3 (1.31) |  | 0.60 (0.19; 1.91) | 0.70 (0.22; 2.24) |
|  | **Drivers** | 280 (0.91) | 4 (1.43) |  | 0.66 (0.24; 1.79) | 0.83 (0.30; 2.28) |
|  | **Cleaners** | 411 (1.34) | 12 (2.92) |  | 1.37 (0.75; 2.49) | 1.53 (0.83; 2.81) |
|  | **Housewives** | 3,564 (11.64) | 100 (2.81) |  | 1.31 (1.01; 1.70) | 0.95 (0.69; 1.29) |
|  | **Students** | 830 (2.71) | 12 (1.45) |  | 0.67 (0.37; 1.21) | 1.28 (0.69; 2.40) |
|  | **Retired** | 4,394 (14.35) | 172 (3.91) |  | 1.85 (1.48; 2.32) | 1.40 (1.06; 1.83) |
|  | **Unemployed** | 661 (2.16) | 6 (0.91) |  | 0.42 (0.18; 0.95) | 0.45 (0.18; 1.11) |
|  | **Other** | 3,847 (12.56) | 70 (1.82) |  | 0.84 (0.63; 1.12) | 0.82 (0.60; 1.12) |
|  | **Military** | 159 (0.52) | 4 (2.52) |  | 1.17 (0.43; 3.21) | 1.93 (0.69; 5.35) |
